# Karyotype analysis and visualization of 45S rRNA genes using fluorescence *in situ* hybridization in aroids (Araceae)

**DOI:** 10.3897/CompCytogen.v9i2.4366

**Published:** 2015-05-11

**Authors:** Prabhu Shankar Lakshmanan, Katrijn Van Laere, Tom Eeckhaut, Johan Van Huylenbroeck, Erik Van Bockstaele, Ludmila Khrustaleva

**Affiliations:** 1 Institute for Agricultural and Fisheries Research (ILVO), Plant Sciences Unit, Applied Genetics and Breeding, Caritasstraat 21, 9090 Melle, Belgium; 2 Department of Plant Production, Faculty of Bioscience Engineering, Ghent University (UGent), Coupure links 653, 9000 Ghent, Belgium; 3 Center of Molecular Biotechnology, Department of Genetics and Biotechnology, Russian State Agrarian University-Timiryazev Agricultural Academy (TIMACAD), 49, Timiryazevskaya str., 127550 Moscow, Russia

**Keywords:** Araceae, B-chromosomes, chromosome formula, cytogenetics, genome size, FISH

## Abstract

Karyotype analysis and FISH mapping using 45S rDNA sequences on 6 economically important plant species *Anthurium andraeanum* Linden ex André, 1877, *Monstera deliciosa* Liebmann, 1849, *Philodendron scandens* Koch & Sello, 1853, *Spathiphyllum wallisii* Regel, 1877, *Syngonium auritum* (Linnaeus, 1759) Schott, 1829 and *Zantedeschia elliottiana* (Knight, 1890) Engler, 1915 within the monocotyledonous family Araceae (aroids) were performed. Chromosome numbers varied between 2n=2x=24 and 2n=2x=60 and the chromosome length varied between 15.77 µm and 1.87 µm. No correlation between chromosome numbers and genome sizes was observed for the studied genera. The chromosome formulas contained only metacentric and submetacentric chromosomes, except for *Philodendron scandens* in which also telocentric and subtelocentric chromosomes were observed. The highest degree of compaction was calculated for *Spathiphyllum wallisii* (66.49Mbp/µm). B-chromosome-like structures were observed in *Anthurium andraeanum*. Their measured size was 1.87 times smaller than the length of the shortest chromosome. After FISH experiments, two 45S rDNA sites were observed in 5 genera. Only in *Zantedeschia elliottiana*, 4 sites were seen. Our results showed clear cytogenetic differences among genera within Araceae, and are the first molecular cytogenetics report for these genera. These chromosome data and molecular cytogenetic information are useful in aroid breeding programmes, systematics and evolutionary studies.

## Introduction

The Araceae (commonly known as aroids) are a very widely distributed monocotyledonous family. Most aroids are tropical and subtropical species while some members are growing in temperate regions. There are about 117 genera and 3300 species (Mayo et al. 1997; Boyce and Croat 2013). The leaves of aroids often show broad netted venation. The inflorescence possesses a dense mass of apetalous flowers on a central ‘spadix’. The flowers are generally covered in a leaf like ‘spathe’, which can be colored or colorless. Because of this attractive feature, aroids are commonly used as ornamentals (cut flowers and pot plants) or for landscaping in more (sub) tropical areas (Chen et al. 2005). However, more molecular cytogenetic information would be very useful for plant systematics and evolutionary studies and in plant breeding programs. In breeding programs, (cyto)genetic information of parent plants can be useful to select suitable parent combinations and to trace parental markers in putative hybrids.

In cytogenetic studies, one of the first goals is chromosome identification and karyotype construction based on microscopic morphological characteristics of the chromosomes. In addition to morphological chromosome features, by molecular cytogenetic techniques such as fluorescence *in situ* hybridization (FISH) and based on DNA sequence information, chromatin regions of individual chromosomes can be addressed (Shubert et al. 2001). FISH has become important for physical mapping of single-copy DNA sequences of interesting genes, e.g. economically important genes relevant for breeding programs. FISH is also particularly valuable for identifying the sites of highly repetitive genes, e.g. rRNA genes, which are difficult to map by other methods (Leitch and Heslop-Harrison 1992). The localization of this repetitive DNA using FISH can play a role in chromosome identification and karyotype analysis. FISH of single-copy DNA sequences and repetitive sequences has become indispensable in map-based cloning and other physical mapping strategies. rRNA genes have been isolated from many different plant species and used as probes for FISH (Schwarzacher 2003). FISH with rRNA genes can also help to detect recent polyploidization (duplication or dysploidy), since the number of 5S rDNA and 45S rDNA sites sometimes doubles with polyploidization (Souza et al. 2010). Up till now, only very little molecular cytogenetic information is known for Araceae. Cusimano et al. (2011) performed a phylogenetic study to infer Araceae chromosome evolution based on molecular data compared with morphological and anatomical data analyses. In their study, Cusimano et al (2011) distinguished 44 clades having morphological or anatomical synapomorphies as well as ecological or geographic cohesion. Chromosome numbers are available for 862 species (26% of the family), ranging from 2n=10 to 2n=168 (Cusimano et al. 2012). Cusimano et al. (2012) suggested an ancestral haploid chromosome number of 16 or 18, rather than the base number of x=7 (Larsen 1969; Marchant 1973) or x=14 (Petersen 1993) previously hypothesized. Few karyotype studies for species distinction and relationship have been reported (Fu-Hua et al. 2001; Chen et al. 2007; Begum et al. 2009; Ghimire et al. 2012). And physical mapping of repetitive sequences such as 45S or 5S rDNA using FISH has only been reported for *Typhonium* Schott, 1829 (Sousa et al. 2014).

In our study, flow cytometric analysis for genome size measurements, karyotype construction, and FISH mapping using 45S rDNA sequences were performed for the first time on the Araceae species *Anthurium andraeanum* Linden ex André, 1877, *Monstera deliciosa* Liebmann, 1849, *Philodendron scandens* Koch & Sello, 1853, *Spathiphyllum wallisii* Regel, 1877, *Syngonium auritum* (Linnaeus, 1759) Schott, 1829 and *Zantedeschia elliottiana* (Knight, 1890) Engler, 1915. These six species were chosen for their economic importance as ornamental species.

## Material and methods

### Plant material

*Anthurium andraeanum* ‘061’ and *Spathiphyllum wallisii* ‘Domino’ were present in the ILVO collection; *Monstera deliciosa* ‘Variegata’, *Philodendron scandens* and *Syngonium auritum* were obtained from the greenhouse of Tsitsin RAS Botanical Garden, Moscow, Russia; *Zantedeschia elliottiana* ‘068’ was provided by Sandegroup, the Netherlands. The plants used in this study are known ornamental cultivars (no hybrids). The plants were grown in greenhouse conditions (20±2 °C; 16 h/day at 30 µmol m^-2^ s^-1^ photosynthetic period, 60±3% relative humidity) in terracotta pots, filled with potting soil (Saniflor®, NV Van ISRAEL, Geraardsbergen, Belgium) and watered two days before collecting the root tips.

### Genome size measurements

Genome size analysis was performed according to Dewitte et al. (2009) using young leaf material. A minimum of 5000 nuclei were analyzed per sample. Obtained data were analyzed using Flomax software on a CyFlow space of PASIII (Partec).

The following reference plants were used: *Pisum sativum* Linnaeus, 1759 ‘Ctirad’ (9.09 pg/2C; Doležel et al. 1998) for *Spathiphyllum wallisii* ‘Domino’; *Solanum lycopersicum* Linnaeus, 1759 ‘Stupické Polní Rané’ (1.96 pg/2C; Doležel et al. 1992) for *Philodendron scandens*, *Syngonium auritum* and *Zantedeschia elliottiana* ‘068’; and *Glycine max* (Linnaeus, 1753) Merrill, 1917, ‘Polank’ (2.5 pg/2C; Doležel et al. 1994) for *Anthurium andraeanum* ’061’ and *Monstera deliciosa* ‘Variegata’. At least three repeats were analyzed. The genome size was calculated based upon peak position ratios of the sample plants and the reference plants. The influence of plant cytosolic compounds on fluorochrome accessibility of nuclear DNA was tested. To this end, we tested the stability of the peak positions of the reference plants, either with or without sample plants, in all tests.

### Chromosome spread preparation

Actively growing root tips were collected. The root tips of *Spathiphyllum wallisii* ‘Domino’ were pretreated in ice-cold (4 °C) water overnight. *Anthurium andraeanum* ‘061’, *Monstera deliciosa* ‘Variegata’, *Philodendron scandens*, *Syngonium auritum*, and *Zantedeschia elliottiana* ‘068’ root tips were pretreated in a α-bromonaphthalin solution overnight at 4 °C. α-Bromonaphthalin solution was prepared dissolving 10 µL of α-bromonaphthalin in 10 mL water. After the pretreatment, the root tips were fixed in Carnoy solution (3:1 absolute ethanol-acetic acid) at least 1 h at room temperature. They were either used immediately or stored at -20 °C until use. The Carnoy solution was removed by washing the root tips three times in tap water for 20 minutes. The root tips were digested in a pectolytic enzyme mixture [0.1% (w/v) pectolyase Y23 (Duchefa, Haarlem, the Netherlands), 0.1% (w/v) cellulase onozuka RS (Duchefa, Haarlem, the Netherlands) and 0.1% (w/v) cytohelicase (Sigma-Aldrich, Steinheim, Germany)] in 10 mM citrate buffer (10mM tri sodium citrate + 10 mM citrate, pH 4.5) at 37 °C for 1 h. Chromosome preparations were made according to the spreading method of Pijnacker and Ferwerda (1984). The best slides were selected under a phase contrast microscope (Leica DM IRB).

### *In situ* hybridization

Plasmid clone pTa71 containing a 9 kb *Eco*RI fragment of the 45S rDNA from *Triticum aestivum* Linnaeus, 1753 (Gerlach and Bedbrook 1979) were used. Isolated pTa71 plasmids were labelled with a Biotin-Nick Translation Kit (Roche Diagnostics Gmbh, Mannheim, Germany) or Digoxigenin-Nick Translation Mix (Roche Diagnostics Gmbh, Mannheim, Germany), respectively, according to manufacturer’s instructions.

Slides were pretreated with 4% (w/v) paraformaldehyde for 10 min at room temperature and air dried after sequential washes in 70% (-20 °C), 90% and 100% ethanol for 3 min each (Leitch and Heslop-Harrison 1994). DNA denaturation and *in situ* hybridization were done according to Schwarzacher and Leitch (1993) and Schwarzacher and Heslop-Harrison (1994). The hybridization mixture was made of 50% (v/v) deionized formamide, 10% (w/v) dextran sulphate, 2x SSC (Saline Sodium Citrate buffer), 0.25% (w/v) sodium dodecyl sulphate and 2 ng/µL labelled DNA. The hybridization mixture was denatured at 80 °C for 5 min and placed on ice for 5 min. After the hybridization mixture (40 µL) was added to the slides, a 5 min denaturation process was carried out at 80 °C. Then the slides were incubated overnight in a humid chamber at 37 °C to hybridize. The slides were washed in 2x SSC at room temperature for 15 min, then transferred to 0.1x SSC at 48 °C for exactly 30 minutes to give a 78% stringent wash (Schwarzacher and Heslop-Harrison 2000). Finally, they were washed again in 2x SSC for 15 min at room temperature. To reduce non-specific binding of antibodies and thus to lower the background fluorescence, 100 µL of 1% TNB [Boeringer blocking reagent in TN buffer (0.1 M Tris-HCl, 0.15 M NaCl, and pH 7.5)] was added to the slides and incubated for 10 min at 37 °C in a humid chamber. Biotin-labelled DNA was detected with 5 µL CY3-conjugated streptavidin and amplified with 1 µL biotinylated goat-antistreptavidin (Vector Laboratories, Burlingame, CA, USA) followed by addition of CY3-conjugated streptavidin. Digoxigenin-labelled probes were detected using FITC conjugated anti-Dig antibody (0.01% FITC in TNB; Roche Diagnostics Gmbh, Mannheim, Germany) from sheep and 1µL anti-sheep FITC from rabbit diluted in TNB. These detection steps were performed at 37 °C in a humid chamber for 1 h. Biotin-labelled DNA was only used for *Zantedeschia elliottiana* ‘068’.

### Microscopy and karyotyping

The slides were counterstained with 1µg/mL 4’,6-Diamidino-2-phenylindole (DAPI) and mounted with Vectashield® (Vector Laboratories, Burlingame, CA, USA). Slides were examined under a Zeiss Axio Imager microscope (Carl Zeiss MicroImaging, Jena, Germany). Images were captured by AxioCam and Axiovision 4.6 software, Zeiss. Karyotype analysis was done on five well-spread, DAPI stained metaphases for *Anthurium andraeanum*, *Monstera deliciosa*, *Philodendron scandens*, *Spathiphyllum wallisii*, *Syngonium auritum* and 10 metaphases for *Zantedeschia elliottiana* using MicroMeasure (Reeves 2001) for Windows, version 3.3. Arm lengths were measured and relative chromosome length (percentage length of the individual chromosome/total length of all chromosomes in the genome at haploid level), and centromeric index (length of short arm divided by total chromosome length X 100) were calculated. The position of the hybridization signal were measured. FISH signal positions were determined analyzing 3 spreads from *Anthurium andraeanum* and *Spathiphyllum wallisii*; 5 spreads from *Monstera deliciosa* and *Philodendron scandens*; 4 for *Syngonium auritum* and 6 for *Zantedeschia elliottiana*. Characterization of chromosome type was done based on centromeric index as mentioned by Levan et al. (1964). Chromosomes were arranged in order of decreasing length. The asymmetry of the karyotype was evaluated according to Paszko (2006). The degree of chromosome compaction [Genome size 1C (Mbp) / mean total chromosome length (µm)] was calculated assuming that it is uniform along the entire chromosome.

## Results

**Figure 1.**
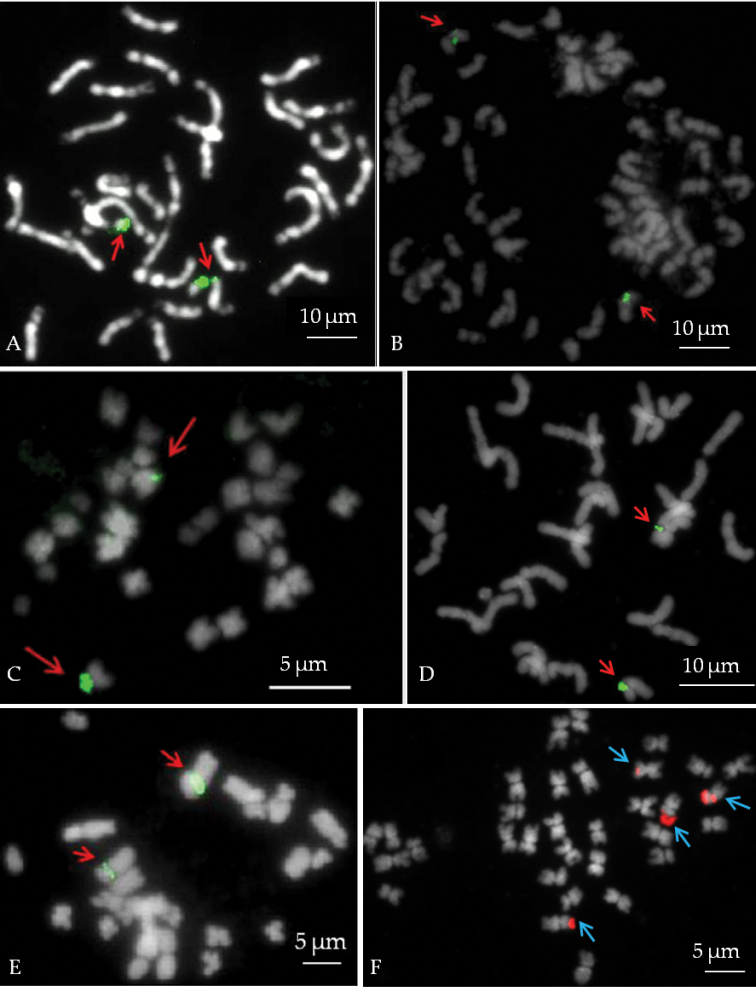
DAPI stained mitotic metaphases with FISH signal: **A** *Anthurium andraeanum* ‘061’ **B** *Monstera deliciosa* ‘Variegata’ **C** *Philodendron scandens* **D** *Spathiphyllum wallisii* ‘Domino’ **E** *Syngonium auritum*; and **F** *Zantedeschia elliottiana* ‘068’. 45S rDNA FISH signals are indicated by arrows. 45S rDNA sites were observed using FITC (green **A–E**) and using CY3 (red **F**).

**Figure 2.**
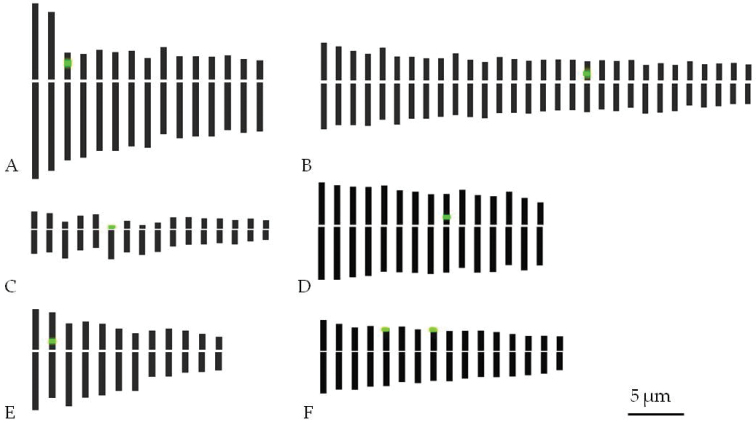
Idiograms with indication of 45S rDNA (green) based on observation: **A** *Anthurium andraeanum* ‘061’ **B** *Monstera deliciosa* ‘Variegata’ **C** *Philodendron scandens* **D** *Spathiphyllum wallisii* Regel ‘Domino’ **E** *Syngonium auritum*; and **F** *Zantedeschia elliottiana* ‘068’.

The results for genome size measurements and karyotype analysis are summarized in Table 1. Metaphases are shown in Fig. 1 and the idiograms in Fig. 2. Flow cytometric analysis showed the small genome size for *Zantedeschia elliottiana*, *Philodendron scandens* and *Syngonium auritum* while for *Spathiphyllum wallisii* the largest genome size (7.39 ± 0.04 pg/1C) was observed. *Monstera deliciosa* (2n=60) had the highest chromosome number. The lowest chromosome number was found in *Syngonium auritum* (2n=24). The total chromosome length at haploid chromosome level varied between 147.14 ± 0.39 µm and 44.64 ± 0.52 µm. Compared to the other species *Anthurium andraeanum* and *Spathiphyllum wallisii* had the biggest chromosomes. *Philodendron scandens* possessed the smallest chromosomes and was the only species that contains subtelocentric and telocentric chromosomes besides metacentric and submetacentric chromosomes (Fig. 2). This is reflected in the asymmetry index (AI) for *Philodendron scandens* (Table 1). The degree of compaction was the highest in *Spathiphyllum wallisii* and the lowest in *Zantedeschia elliottiana*.

**Table 1.** Summary of genome size and karyotypic data for *Anthurium andraeanum* ‘061’, *Monstera deliciosa* ‘Variegata’, *Philodendron scandens*, *Spathiphyllum wallisii* ‘Domino’, *Syngonium auritum* and *Zantedeschia elliottiana* ‘068’. Data are averages ± SE. (n=5, except for *Zantedeschia elliottiana* n=10).

	*Anthurium andraeanum*	*Monstera deliciosa*	*Philodendron scandens*	*Spathiphyllum wallisii*	*Syngonium auritum*	*Zantedeschia elliottiana*
Genome size (pg/1C)	5.27 ± 0.08	6.36 ± 0.22	1.74 ± 0.01	7.39 ± 0.04	2.60 ± 0.04	1.35 ± 0.01
Chromosome number	2n=2x=30	2n=2x=60	2n=2x=32	2n=2x=30	2n=2x=24	2n=2x=32
Chromosome formula^z^	3m+12sm	26m+4sm	10m+2sm+3st+1t	15m	8m+4sm	16m
Total chromosome complement (µm)^y^	132.72 ± 1.39	147.14 ± 0.39	44.64 ± 0.52	107.97 ± 0.58	64.88± 1.25	73.19 ± 0.99
Length of the longest chromosome (µm)	15.77 ± 1.72	7.76 ± 0.99	3.81 ± 0.75	8.58 ± 0.02	8.71 ± 1.91	6.51 ± 2.10
Length of the shortest chromosome (µm)	6.20 ± 0.10	3.35 ± 0.40	1.87 ± 0.33	5.64 ± 1.35	3.07 ± 0.57	3.01 ± 0.85
Asymmetry index (AI)	5.49	1.90	6.58	0.70	4.31	0.90
Degree of compaction (Mbp/µm)	38.52 ± 0.19	41.98 ± 0.29	37.74 ± 0.39	66.49 ± 0.37	38.85 ± 2.02	17.84 ± 0.59
45S rRNA gene chromosome number(s)	3	19	6	9	2	5, 8

z m – metacentric; sm – submetacentric; st – subtelocentric; t – telocentric

y Total chromosome length at haploid level

**Figure 3.**
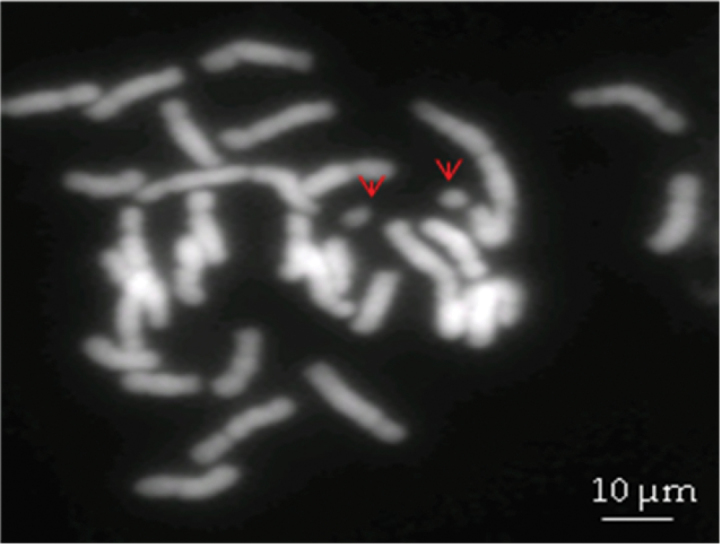
DAPI stained chromosome spreads of *Anthurium andraeanum* ‘061’ with presumable B-chromosomes (indicated by arrows).

B-chromosome-like structures were observed in *Anthurium andraeanum* metaphase spreads (Fig. 3). Approximately 19.75% cells possessed two B-chromosomes-like structures, 34.57% spreads showed one and 45.68% showed none. The size of B-chromosome-like structures was 3.32 ± 0.12 µm (measurement on 15 B chromosome like structures), which is about 1.87 times less than the size of the shortest chromosome in the complement (6.20 ± 0.10 µm). B-chromosome structures were not seen in any other of the studied plants.

45S rRNA genes were visualized using FISH (Fig. 1). In all genera, two 45S rDNA sites were visualized except in *Zantedeschia elliottiana* which had four 45S rDNA sites (Table 1). 45S rDNA sites were seen in a distal position of *Anthurium andraeanum* and *Zantedeschia elliottiana* short arms and on the proximal position of the short arms in other species (Figs 1 and 2). In *Philodendron scandens*, signals were observed at the terminal position of the telocentric chromosome (Fig. 1 C).

## Discussion

The success of interspecific or intergeneric crosses using traditional breeding mainly depends on how closely the parental species are (cyto) genetically related. Moreover, differences between parent plants concerning chromosome number, genome size and morphology of pairing chromosomes decide the fate of hybrid chromosome pairing during meiosis. According to Cusimano et al. (2011), the six genera we tested belong to different groups. Among them *Anthurium* Schott, 1829, *Monstera* Adanson, 1763 and *Spathiphyllum* Schott, 1832 are closer to each other by sharing some morphological and anatomical features while *Philodendron* Schott, 1829, *Syngonium* Schott, 1829 and *Zantedeschia* Sprengel, 1826 are very distantly related. In our study, we also detected many cytogenetic differences among them.

The first things we noticed were different chromosome numbers and genome sizes among the six genera. The commonly known chromosome number for *Anthurium andraeanum* is 2n=30 (Marutani et al. 1993; Cusimano et al. 2011) which is in agreement with our results. However, there is also a report of 2n=32 for *Anthurium andraeanum* (Sheffer and Croat 1983). An equal chromosome number of 30 was reported for *Spathiphyllum* (Marchant 1973; Cusimano et al. 2011) and confirmed in our study. Also for *Philodendron scandens* and *Zantedeschia* our results are in agreement with earlier findings of 2n=32 (Marchant 1971a; 1971b). The chromosome number (2n=24) for *Syngonium auritum* in our study and *Syngonium wendlandii* Schott, 1858 are similar. However, other *Syngonium* species have varying chromosome numbers (Marchant 1971b; Cusimano et al. 2011). Different authors have reported different *Monstera deliciosa* chromosome numbers (Petersen 1989). Our counts for *Monstera deliciosa* ’Variegata’ (2n=60) agree with the counts of Marchant (1970) and Cusimano et al. (2012). The varying chromosome numbers within genera might be explained by aneuploid derivations such as chromosome losses or gains after meiotic irregularities leading to the formation of aneuploid gametes (Petersen 1989; Sousa et al. 2014). We might conclude that the higher chromosome numbers in *Monstera deliciosa* compared to other Araceae plants, might be due to either an ancient polyploidization origin of the genus or a high basic chromosome number.

Araceae genome sizes are described to vary between 0.33 (*Lemna* Linnaeus, 1753; Wang et al. 2011) and 24.05 pg/1C (*Zamioculcas* Schott, 1856; Zonneveld et al. 2005). The six genera we analyzed also showed significant differences in genome sizes. *Anthurium*, *Spathiphyllum* and *Monstera* had higher genome sizes. For *Anthurium* and *Spathiphyllum*, our results were consistent with earlier reported genome sizes of 4.49 pg/1C for *Anthurium andraeanum* (Bliss et al., 2012), and 7.11 pg/1C for *Spathiphyllum* (Zhao et al., 2012). For *Zantedeschia elliottiana*, the total genomic content calculated in our study (1.35 pg/1C) was clearly higher than the 0.59 pg/1C mentioned by Ghimire et al. (2012). Therefore, we also repeated flow cytometrical analysis using *Pisum sativum* L. ‘Citrad’ as the reference plant. This additional analysis confirmed *Zantedeschia elliottiana* genome size as 1.30 pg/1C.

Although *Spathiphyllum wallisii* ‘Domino’ had the largest genome size, the total chromosome complement was lower than in *Anthurium andraeanum* and *Monstera deliciosa*. *Zantedeschia elliottiana* had a higher chromosome length than *Philodendron scandens* and *Syngonium auritum* although its DNA content was lower. A direct correlation between total chromosome complement and genomic content is reported (Cerbah et al. 2001; Zonneveld 2004). However, also negative correlations have been reported (Van Laere et al. 2008).

Karyotypic symmetry varies according to the presence of different chromosome types. A symmetrical karyotype mainly possesses metacentric and submetacentric chromosomes of approximately equal size whereas asymmetric karyotypes arise by shifts in centromeric position towards the telomere, and/or by addition or deletion of chromatin in some chromosomes, which gives rise to size differences (Stebbins 1971). The most common chromosome morphology type was metacentric, followed by submetacentric. Subtelocentric and telocentric chromosomes were only observed in *Philodendron scandens*, which showed also the highest asymmetry index. The karyotype we found for *Anthurium andraeanum* is comparable to the one published by Kaneko and Kamemoto (1979) for *Anthurium warocqueanum* Moore, 1878: 2 pairs of large chromosomes, 1 pair of satellite chromosomes and 12 pairs of small chromosomes. However, the size of the chromosomes differed between both species. Additionally, the choice of the pretreatment, fixating agents and chromosome preparation techniques considerably influence the chromosome structure (Sharma and Bhattacharyya 1961).

*Zantedeschia elliottiana* karyotypic data differed from those published by Ghimire et al. (2012). Various factors might affect karyotypic results of which chromosome fixation, slide preparation or chromosome staining method are very important and different in the study of Ghimire et al. (2012) compared to our study. Moreover, DAPI staining (fluorescent) is preferred over other staining methods as it can provide a stronger signal (Maluszynska 2003; Van Laere et al. 2008).

Supernumerary or putative B chromosomes have been reported in some gerera of Araceae, such as *Anthurium* (Sharma and Bhattacharyya 1961; Kaneko and Kamemoto 1979; Marutani et al. 1993), *Apoballis* Schott, 1858, *Arisaema* Martius, 1831, *Asterostigma lividium* (Loddiges, 1830) Engler, 1930 *Philodendron radiatum* Schott, 1853, *Piptospatha burbidgei* (Brown, 1882) Hotta, 1965, *Schismatoglottis* Zollinger & Moritzi, 1846 and *Typhonium* Schott, 1829 (Sousa et al. 2014). The size of the B-chromosomes in *Anthurium ochranthum* Koch, 1853 was smaller than the smallest chromosome in the karyotype while in *Anthurium garagaranum* Standley, 1940 the B-chromosome had the same size as the smallest regular chromosome (Marutani et al. 1993). However, none of these studies used meiotic analysis for a more detailed understanding. B-chromosomes are unnecessary components in the karyotypes of some plants, fungi and animal species. They are present in some individuals of a population and absent in others. They do not pair or recombine with any chromosomes (A-chromosomes) of the standard diploid (or polyploid) at meiosis and their inheritance is non-mendelian and irregular (Jones and Houben 2003). In our study, we observed one or two B-chromosome-like structures in almost 50% of the spreads of *Anthurium andraeanum*. Our experiments, of course, are insufficient to establish the presence of B chromosomes. Meiotic stage analyzes are needed to confirm their presence and to exclude that they are broken chromosome arms or satellites.

The six genera we analyzed showed different chromosome condensation indices. DNA condensation variation is also described in other plant genera. For instance, in onion condensation is six times higher than in tomato (Khrustaleva and Kik 2001). Van Laere et al. (2008) and Lysak et al. (1999) even reported varying genomic condensation differences among genera and subspecies as well as among accessions. They also proposed the geographical origin of the plants, even within species, as a probable cause for the differences. However, there is no clear proof yet that geographical origin plays a major role in DNA condensation. In our studies, *Zantedeschia elliottiana*, having less condensed chromosomes, is the only South African species, whereas all other genera in this study originated in tropical America.

Finally, we applied FISH in order to localize the 45S rDNA chromosome markers. No secondary constriction could be distinguished in the DAPI stained spreads. DAPI binds to AT rich heterochromatic regions, whereas the nucleolus organizing region (NOR) is composed of GC rich tandem repeats (Lima-de-Faria 1976). Generally, 45S rDNA is associated with a NOR in eukaryotes and NOR is often positioned with a secondary constriction such as satellites (Roa and Guerra 2012). Sometimes, these secondary constrictions are lost during slide preparation. There are few reports of 45S rDNA signal without visible satellites (Ricroch et al. 1992, Van Laere et al. 2008). In our study, 45S rDNA signals were observed in the short chromosome arms as it was reported by Lima-de-Faria (1976). All 45S signals were localized on the short arms; for *Anthurium andraeanum* at intercalary position, for *Monstera deliciosa*, *Philodendron scandens*, *Spathiphyllum wallisii* and *Syngonium auritum* near the centromere, and for *Zantedeschia elliottiana* at the distal end. Five of the six investigated genera had 2 45S rDNA signals. Only *Zantedeschia* had 4 45S rDNA signals. This increase of rDNA sites might indicate an ancient polyploidization, although they had a similar chromosome amount as e.g. *Spathiphyllum* which had only 2 45S rDNA signals. Known polyploid angiosperms commonly show an increased number of rDNA sites. Alternative explanations involve jumping NOR regions, perhaps mediated by transposable elements (Raskina et al. 2008).

In conclusion, our results give a clear first view on the cytogenetic differences among six genera within Araceae which is a valuable addition to the phylogenetic differences demonstrated by Cusimano et al. (2011; 2012). All these data constitute a basic knowledge of genetic resources, resulting in an advantageous feature for facilitating molecular approaches to study taxonomic relationships, evolutionary events such as past chromosome number changes, chromosomal aberrations and cellular functions. Moreover, for plant breeding purposes, the choice of species for interspecific hybridization is sometimes critical. When parental species differ sufficiently in nuclear DNA content and chromosome morphology, it is easier to detect interspecific hybrids by intermediate values of DNA or by FISH techniques. Cytogenetic markers can then be used to trace back chromosomal behaviour of parental plants in the hybrids. On the other hand, when DNA differences are too large, e.g. very different karyotype, large sequence divergence, interspecific crosses will not be successful. Therefore, our results can be used by breeders to select suitable parents for interspecific or intergeneric crosses in aroids in future aroid breeding programs and can be a first step for establishing Genomic *In Situ* Hybridization (GISH) in Araceae. Finally, the physical localization of the rRNA genes can provide a first link between (future) physical and genetic maps, which can lead to a better understanding of the genetic structure of the Aroids.
